# Genetic dissection of ozone tolerance in rice (*Oryza sativa* L.) by a genome-wide association study

**DOI:** 10.1093/jxb/eru419

**Published:** 2014-11-04

**Authors:** Yoshiaki Ueda, Felix Frimpong, Yitao Qi, Elsa Matthus, Linbo Wu, Stefanie Höller, Thorsten Kraska, Michael Frei

**Affiliations:** ^1^Institute of Crop Science and Resource Conservation (INRES), University of Bonn, Karlrobert-Kreiten Strasse 13, 53115 Bonn, Germany; ^2^Key Laboratory of Crop Genetics & Physiology of Jiangsu Province, Yangzhou University, Yangzhou 225009, PR China; ^3^Campus Klein-Altendorf, University of Bonn, Klein-Altendorf 2, 53359 Rheinbach, Germany

**Keywords:** Biomass production, EREBP, genome-wide association study (GWAS), leaf symptoms, lignin, ozone, rice (*Oryza sativa* L.), RING protein.

## Abstract

Tropospheric ozone causes various negative effects on plants and affects the yield and quality of agricultural crops. Here, we report a genome-wide association study (GWAS) in rice (*Oryza sativa* L.) to determine candidate loci associated with ozone tolerance. A diversity panel consisting of 328 accessions representing all subgroups of *O. sativa* was exposed to ozone stress at 60 nl l^–1^ for 7h every day throughout the growth season, or to control conditions. Averaged over all genotypes, ozone significantly affected biomass-related traits (plant height –1.0%, shoot dry weight –15.9%, tiller number –8.3%, grain weight –9.3%, total panicle weight –19.7%, single panicle weight –5.5%) and biochemical/physiological traits (symptom formation, SPAD value –4.4%, foliar lignin content +3.4%). A wide range of genotypic variance in response to ozone stress were observed in all phenotypes. Association mapping based on more than 30 000 single-nucleotide polymorphism (SNP) markers yielded 16 significant markers throughout the genome by applying a significance threshold of *P*<0.0001. Furthermore, by determining linkage disequilibrium blocks associated with significant SNPs, we gained a total of 195 candidate genes for these traits. The following sequence analysis revealed a number of novel polymorphisms in two candidate genes for the formation of visible leaf symptoms, a *RING* and an *EREBP* gene, both of which are involved in cell death and stress defence reactions. This study demonstrated substantial natural variation of responses to ozone in rice and the possibility of using GWAS in elucidating the genetic factors underlying ozone tolerance.

## Highlight

A genome-wide association study with rice revealed a wide range of natural variation to ozone stress and identified candidate loci affecting nine traits of physiological and agronomical importance.

## Introduction

Due to anthropogenic gas emissions, the tropospheric ozone concentration is increasing and negatively affects natural vegetation and crop production (Ainsworth *et al.*, 2012). Ozone is known to reduce photosynthetic rates (Chen *et al.*, 2011) and induce cell death (Kangasjärvi *et al.*, 2005) and affects numerous metabolic pathways (Frei *et al.*, 2010), thus decreasing crop yields (Feng and Kobayashi, 2009) and changing crop quality (Wang and Frei, 2011). A steep increase in tropospheric ozone has been observed mainly in Asian countries (Lei *et al.*, 2013), where rice is the staple food. It is estimated that rice grain yields are already being affected in Asian countries at present ozone levels. Teixeira *et al.* (2011) estimated yield losses of 18 million and 11 million t of rice per year in India and China, respectively, which corresponds to more than 5% of relative loss due to increased tropospheric ozone.

Some typical symptoms of ozone stress in plants are directly related to crop quality and yield: (i) chlorosis and pale colour of leaves; (ii) necrotic dark brown spots or dead regions on leaves; and (iii) reduced growth rate and a stunted phenotype, leading to reduced yield. Among those traits, necrotic dark brown spots are closely related to acute ozone stress and are caused either by direct oxidative damage or by programmed cell death, which involves plant hormonal pathways (Kangasjärvi *et al.*, 2005). Generally, the correlation between the above-mentioned traits is not very pronounced, suggesting that they are under independent genetic control. For example, little correlation was observed between the extent of leaf damage and growth reduction (Frei *et al.*, 2008) or grain yield (Sawada and Kohno, 2009) in screening experiments with rice.

Genotypic variation in adaptation to ozone has been reported for a number of crop species such as rice, snap bean, and wheat (Flowers *et al.*, 2007; Frei *et al.*, 2008; Feng *et al.*, 2011). However, only a few studies have attempted to dissect and use this genetic variability in ozone tolerance for molecular breeding of ozone-tolerant crop genotypes. A number of genetic mapping studies have employed bi-parental mapping populations to identify quantitative trait loci (QTL) for ozone tolerance (Frei *et al.*, 2008; Brosché *et al.*, 2010; Street *et al.*, 2011; Tsukahara *et al.*, 2013). In our previous study, such a QTL-based approach was successfully employed in developing rice genotypes with superior quality traits (Frei *et al.*, 2011) and biomass production (Wang *et al.*, 2014) under ozone stress. The shortcoming of such classical QTL studies is that they use bi-parental mapping populations, thus covering only a narrow genetic variability. Moreover, the resolution of mapping is limited by the number of genetic recombination events occurring in the mapping populations (Flint-Garcia *et al.*, 2003).

More recently, genome-wide association study (GWAS) has been emerging as a powerful tool to dissect a much broader genetic variability for important traits in crops (Brachi *et al.*, 2011). This method employs populations of unrelated individuals representing a broad genetic variability, and abundant single-nucleotide polymorphisms (SNPs) are usually used as genetic markers. Since such populations reflect the diverse evolutionary recombination events of a species, high-resolution mapping is possible depending on the extent of linkage disequilibrium (LD) in the population. In the case of rice, which was developed into a domesticated crop by self-pollination plus forced pollination by humans, GWAS has been successfully conducted with a limited number of markers due to relatively slow LD decay (half decay is achieved in around 100–200kb, compared with 0.5–7kb in the outcrossing crop maize; Gupta *et al.*, 2005), while achieving high resolution. Another advantage of using rice as a model plant is that it has a relatively small genome size, which reduces the number of necessary markers. Zhao *et al.* (2011) conducted a GWAS analysis using 413 rice genotypes from most of the rice-growing areas in the world, based on a 44 000-SNP genotyping array, followed by mapping for 34 agronomically relevant phenotypic traits. They provided evidence for the suitability of their population for GWAS by identifying significant marker associations near known genes affecting certain traits such as plant height. The population, known as the ‘diversity panel’, can thus be used for the mapping of hitherto unknown genes.

Here, we report the first GWAS for ozone tolerance in any agricultural crop using a panel covering a broad genetic diversity and representing all subpopulations of rice. Our aims were: (i) to gain insight into the extent of genetic variability of ozone tolerance in rice; (ii) to dissect this genetic variability into distinct loci; and (iii) to identify possible candidate genes underlying these loci.

## Materials and methods

### Plant materials and growth condition

The experiment was conducted in a greenhouse at the University of Bonn, Germany, from May to November 2013. A mapping population consisting of 328 rice cultivars was obtained from the International Rice Research Institute (The Philippines). The seeds were germinated in the dark for 3 d at 28 °C and then transferred to a greenhouse under natural light. Three-week-old seedlings were transplanted into 2×6 m ponds filled with soil (a local luvisol: 16% clay, 77% silt, 7% sand, 1.2% organic carbon, pH 6.3; Schneider, 2005) at 16.5×19cm spacing. A constant water level of at least 3cm was maintained from 10 d after transplanting throughout the growth season. Each of the six plots contained one replicate of all 328 cultivars in a completely randomized distribution. The plots were randomly assigned to ozone and control treatments, and open-top chambers (height 1.3 m) were built around all plots to ensure an identical microclimate. To avoid nutrient limitations, 107g of K_2_SO_4_ and 98g of Ca(H_2_PO_4_)_2_ were applied to each plot as basal fertilizer at the beginning of the season, and 155g of urea was applied in three splits during the season. Temperature, air humidity, and CO_2_ concentration were constantly monitored at 12min intervals using sensors installed at 2 m height in the greenhouse. The average temperature was 25/19 °C (day/night), the average humidity was 60/80% (day/night), and the average CO_2_ concentration was 460/600 ppm (day/night). Supplementary lighting was installed above the plots to ensure a minimum light intensity of 12.5 klux even on cloudy days. Water was removed from the ponds in week 19, and the plants were harvested in week 21. Panicles were separated from the shoots and dried at 50 °C for at least 72h to complete dryness. The shoot samples were dried for 10 weeks in the greenhouse until they reached a constant moisture content of around 11% and then weighed.

### Ozone treatment

Five weeks after transplanting, ozone fumigation was initiated to induce chronic stress at a target level of 60 ppb for 7h every day. Comparable concentrations are already being observed in some areas and are expected to be reached in many countries in the future (Lelieveld and Dentener, 2000; Yamaji *et al.*, 2006). Ozone was produced using custom-made ozone generators (UB 01; Gemke Technik GmbH, Ennepetal, Germany) with dried air passing through silica gels as input. Ozone output was regulated by an ozone monitor (K 100W; Dr A. Kuntze GmbH, Meerbusch, Germany) with an ozone sensor (GE 760 O3; Dr A. Kuntze GmbH) placed inside the chambers. The generated ozone was blown into a central pipe running above the plant canopy, from which three parallel perforated side pipes for ozone distribution branched off at a distance of 40cm from each other. The pipes were calibrated for even ozone distribution prior to transplanting of rice seedlings using a handheld ozone monitor (series 500; Aeroqual Ltd, Auckland, New Zealand). The fumigation lasted from 9:00 until 16:00 each day for 15 weeks until the end of the growth season. During the growth season, acute ozone stress was applied three times in weeks 8, 10, and 14 after transplanting. The average concentration of acute stress was 150 ppb and it lasted for 7h (9:00 to 16:00). The ozone concentration was constantly monitored by the handheld ozone monitor placed within the canopy during the fumigation. The average ozone concentration recorded was 63 ppb in the ozone treatment (excluding the episodes of acute stress), while in the control the concentration was 12 ppb on average.

### Plant phenotyping

Visible leaf symptoms and SPAD values were measured in week 12 after transplanting (i.e. after two applications of acute ozone stress). Tiller number and plant height were measured during the harvesting. Shoot dry weight (DW), grain yield components, and lignin content were measured after the end of the season.

A modified leaf bronzing score (LBS) (Wissuwa *et al.*, 2006) was assigned to each plant to evaluate leaf symptoms, and ranged from 0 to 10 according to the following criteria after evaluating all the leaves: 0, no ozone-induced symptoms in any of the leaves; 2, some symptoms on a few leaves; 4, easily visible symptoms on a few leaves; 6, moderate to severe symptoms on many leaves; 8, severe symptoms on many leaves; 10, whole plant severely damaged. Most of the symptoms began to emerge after the episodes of acute ozone exposure.

SPAD values were measured using a SPAD 502 instrument (Konica Minolta, Osaka, Japan). Three points were measured at 20cm distance from the tip of the second youngest fully expanded leaf of each plant and the average of the three points was determined.

Thousand-kernel weight (TKW), total panicle weight (TPW), and single panicle weight (SPW) were measured after completely drying the panicles. Twenty randomly chosen grains were weighed from the dried panicles and the value was multiplied by 50 to obtain the TKW. Next, TPW and panicle number were measured, and SPW was obtained by dividing TPW by the number of panicles. Some accessions did not reach grain maturity or showed constitutively high spikelet sterility under the climatic conditions of this study and thus showed very high variability in the data. Therefore, 63 accessions (10 ADMIX, four AROMATIC, three AUS, 26 IND, four TEJ, and 16 TRJ) with less than 10g of TKW were removed from the analysis for grain-related traits (TKW, TPW, and SPW) to only analyse filled grains. A further eight accessions (one ADMIX, six AUS, and one IND) were removed for TPW and SPW because of high grain shattering.

Lignin content was measured in the third youngest fully expanded leaves from the main culm. The leaf samples were dried and pulverized, and the lignin content was determined spectrophotometrically according to Suzuki *et al.* (2009) based on the thioglycolic acid method. The pulverized samples were dried at 60 °C and extracted with water and methanol to obtain cell wall fractions. Then the samples were digested with thioglycolic acid to obtain the thioglycolic acid–lignin (TGAL) complex. Finally, the TGAL was dissolved in 1M NaOH and the concentration was determined based on a standard curve using standard lignin (Sigma, St Louis, USA) dissolved into 1M NaOH. The final lignin content was expressed as a percentage on a DW basis.

### Data analysis

A mean value of three biological replicates was used for the analysis (Supplementary Table S1 at *JXB* online). To remove extreme values in each trait, data points that did not fall into the range of [mean of all accessions±3 standard deviations (SDs)] were removed from the dataset prior to the mapping. This procedure eliminated 32 of the 3075 total data points. A square-root transformation was conducted for LBS prior to the mapping, which showed skewed distribution in the original dataset. One-way analysis of variance (ANOVA) tests for subpopulation comparison were conducted with IBM SPSS version 21 (IBM Corp., Armonk, USA), applying a general linear model (GLM) and Tukey’s HSD for the post-hoc test. Two-way ANOVA tests were applied to analyse the effects of treatment and genotype, and the interaction of both on the phenotypic traits using a GLM. Association mapping was conducted using TASSEL 3.0 (Bradbury *et al.*, 2007) based on the SNP marker data, kinship matrix, and principal component analysis (PCA) matrix described by Zhao *et al.* (2011). This SNP array provides around one SNP every 10kb, covering all 12 chromosomes of rice. SNPs that showed a minor allele frequency (MAF) of <10% in our population were removed to decrease overestimation of the effect of SNPs with a low MAF (Brachi *et al.*, 2011). The resultant number of SNPs was 32 175. A mixed linear model (MLM) was used to calculate the association in all analyses, incorporating both PCA and kinship data. MLM was applied using the default settings (P3D for variance component analysis, compression level set to optimum level). The significance threshold for significantly associated markers was set to *P*<0.0001 (i.e. –log_10_ *P*>4.0). LD analysis was conducted using Haploview 4.2 (Barrett *et al.*, 2005). An LD block was created when the upper 95% confidence bounds of *D’* value exceeded 0.98 and the lower bounds exceeded 0.70 (Gabriel *et al.*, 2002). LD blocks harbouring significant SNPs were then defined as the candidate loci. The genes located in these loci were collected. The gene annotation, closest *Arabidopsis* homologue, and gene ontology (GO) were obtained from the MSU rice genome database (http://rice.plantbiology.msu.edu/, accessed March 2014).

### Candidate gene sequencing and analysis

DNA sequencing of candidate genes was conducted as follows. First, genomic DNA from selected lines was extracted from seeds using a PeqGold plant DNA extraction kit (Peqlab, Erlangen, Germany). The region of interest was amplified by PCR with the following setup: 15 μl of GoTaq Green Master Mix (Promega, Mannheim, Germany), 0.6 μl of each primer (10 μM), 1.5 μl of dimethyl sulfoxide, 10.3 μl of water, and 2 μl of extracted DNA. The following conditions were used for amplification of *EREBP*/*RING* respectively: 95 °C for 2min and 32 cycles of 95 °C for 30 s, 57/55 °C for 30 s, and 72 °C for 2/1.5min, followed by an additional 72 °C extension for 5min. The primer sequences were 5′-AGCCAGCGACTGTGCCAATGTAC-3′ (forward) and 5′-TAATGTCTTCAGCAGTTCAGCCGGAG-3′ (reverse) for *EREBP*, and 5′-CCAAAACCCCCAAAAGCCAATG-3′ (forward) and 5′-ACCACACATCCCTTAGCAATACAC-3′ (reverse) for *RING*. The amplified DNA was purified after gel electrophoresis using a FastGene Gel/PCR Extraction kit (Nippon Genetics, Tokyo, Japan). The purified DNA was subjected to cycle sequencing using one of the primers used for the PCR. Sequences were compared and analysed using MEGA5 software (Tamura *et al.*, 2011). The protein motifs were searched using the InterPro database (https://www.ebi.ac.uk/interpro/, accessed September 2014). Additional genomic sequences of *EREBP* and *RING* were obtained from the TASUKE rice genome browser (http://rice50.dna.affrc.go.jp/, accessed September 2014), where the genomic sequences of 26 accessions from our mapping population were available (Kumagai *et al.*, 2013).

## Results

### Ozone effect and genotypic variation in phenotypic traits

**Fig. 1.**
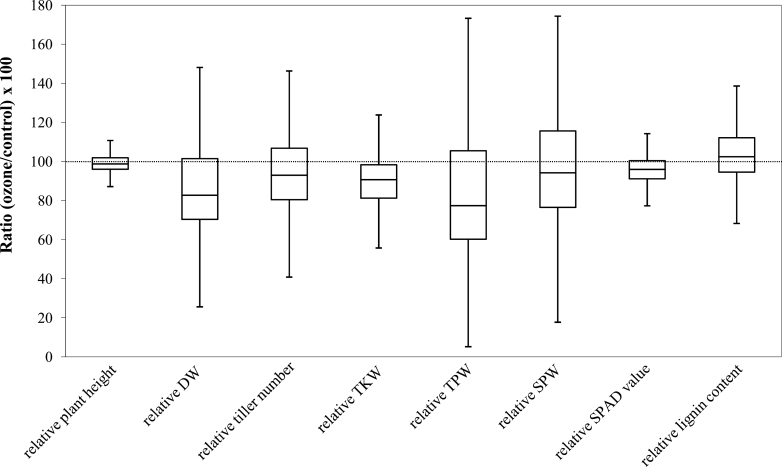
Box plots for relative phenotypic values. The median of each trait is shown as the horizontal bar in the box, and the upper and lower sides of a box represent the first and third quartile values of the distribution, respectively. Whiskers extended to 1.5 times the interquartile range (box size) or to the maximum/minimum values.

We tested the effect of ozone on nine traits, including leaf cell death as represented by LBS; growth parameters such as plant height, shoot DW, and tiller number; grain yield component parameters such as TKW, TPW, and SPW; and biochemical parameters such as chlorophyll content (SPAD value) and foliar lignin content. We also analysed foliar lignin content as an agronomically important parameter, which may represent apoplastic stress, since the coupling of monolignol molecules requires the oxidation of hydroxyl group and therefore is highly dependent on the apoplastic redox status (Frei, 2013). ANOVA analysis (Table 1) demonstrated that all of these traits were significantly affected by the ozone concentration employed, i.e. 60 ppb for 7h daily plus three additional episodes of 150 ppb for 1 d. In plant height, shoot DW, SPW, and SPAD value, we also observed significant interaction between genotype and treatment (G×T). On average, plant height decreased by 1.0%, DW decreased by 15.9%, tiller number decreased by 8.3%, TKW decreased by 9.3%, TPW decreased by 19.7%, SPW decreased by 5.5%, SPAD value decreased by 4.4%, and lignin content increased by 3.4%. Box plots for relative phenotypic values [e.g. relative plant height=(plant height_ozone_/plant height_control_)×100] indicated substantial genotypic variation in ozone responses, which was particularly pronounced in the case of relative DW, relative TPW, and relative SPW (Fig. 1). Growth parameters and grain yield components mostly correlated significantly with each other, and LBS showed a significant correlation with relative tiller number (Table 2). We also compared the ozone response among the five subpopulations (*aromatic*, *aus*, *indica*, temperate *japonica*, and tropical *japonica*) plus the admixed group, which cannot be clearly assigned to any of these subpopulations (Zhao *et al.*, 2010) (Supplementary Fig. S1 and Supplementary Table S2 at *JXB* online). Subpopulations *indica* and temperate *japonica* showed a significantly lower LBS than the other subpopulations. Constitutive lignin content and relative lignin content also showed significant differences among the subpopulations.

**Table 1.** Effect of ozone stress on phenotypic valuesThe phenotypic values under control and ozone are shown. The phenotypic value is the mean of all accessions. ANOVA tests were performed with treatment, genotype, and treatment×genotype as fixed values. *, *P*<0.05; **, *P*<0.01; ***, *P*<0.001. NS, not significant; NA, not analysed.

Phenotype	Control	Ozone	Treatment	Genotype	G×T
LBS	0.0	1.72	***	***	NA^*a*^
Plant height (cm)	181.3	179.4	***	***	*
Shoot DW (g)	43.15	36.27	***	***	***
Tiller number	8.08	7.41	***	***	NS
TKW (g)	20.39	18.50	***	***	NS
TPW (g)	19.83	15.92	***	***	NS
SPW(g)	2.18	2.06	**	***	**
SPAD value	39.21	37.50	***	***	**
Lignin content (%)	1.74	1.80	**	***	NA^*b*^

^*a*^ G×T effect in LBS was not analysed since none of the control plants showed any symptoms.

^*b*^ G×T effect in lignin content was not analysed since there were no biological replicates.

**Table 2.** Correlation coefficient and P value of pair-wise comparison of each phenotypeThe lower triangle shows the Pearson’s correlation coefficient of each pair-wise phenotype comparison. The upper triangle shows the *P* value of the correlation. *P* values were determined by a two-tailed Student’s *t*-test. Data for flowering time in Arkansas (in 305 accessions) were adopted from Zhao *et al.* (2011), where the same mapping population was used.

	LBS	Relative plant height	Relative shoot DW	Relative tiller number	Relative TKW	Relative TPW	Relative SPW	Relative SPAD value	Lignin content	Relative lignin content	Flowering time in Arkansas
LBS	1.00	0.114	0.189	<0.001	0.279	0.545	0.437	0.067	0.182	0.113	0.008
Relative plant height	–0.09	1.00	<0.001	0.056	0.054	0.008	<0.001	0.890	0.095	0.705	0.242
Relative shoot DW	–0.07	0.23	1.00	<0.001	<0.001	<0.001	<0.001	0.970	0.484	0.058	0.300
Relative tiller number	–0.18	0.11	0.68	1.00	0.007	<0.001	<0.001	0.866	0.270	0.189	0.028
Relative TKW	0.07	0.12	0.21	0.16	1.00	<0.001	<0.001	0.334	0.883	0.825	0.353
Relative TPW	–0.04	0.17	0.62	0.56	0.33	1.00	<0.001	0.751	0.608	0.329	0.026
Relative SPW	–0.05	0.25	0.30	0.23	0.42	0.75	1.00	0.582	0.504	0.669	0.461
Relative SPAD value	–0.10	–0.01	0.00	–0.01	–0.06	–0.02	0.03	1.00	0.654	0.197	0.122
Lignin content	0.07	–0.09	–0.04	–0.06	–0.01	–0.03	–0.04	–0.02	1.00	<0.001	0.489
Relative lignin content	0.09	0.02	–0.10	–0.07	–0.01	–0.06	–0.03	–0.07	–0.52	1.00	0.960
Flowering time in Arkansas	0.15	–0.07	–0.06	–0.13	–0.06	–0.14	–0.05	0.09	0.04	0.00	1.00

### Association mapping

We conducted association mapping to identify loci underlying the genetic regulation of the traits mentioned above. We applied an MLM on all datasets, which takes into account the population structure and therefore renders fewer false positives compared with a GLM (Larsson *et al.*, 2013). We set a threshold of –log_10_ *P*>4.0 as a significant association, as adopted in other studies using the same mapping population (Famoso *et al.*, 2011; Zhao *et al.*, 2011). The threshold of –log_10_ *P*>4.0 was also derived from the quantile–quantile (QQ) plots, since most of the upward deviation from the linear line occurred at around –log_10_ *P*=4.0, which presumably indicates true positives. For all traits analysed, we identified 16 SNP markers that satisfied this threshold, being distributed throughout the rice genome (Supplementary Table S3 at *JXB* online). As an alternative approach we also compiled the top 50 SNPs in each trait (i.e. SNPs showing the 50 highest –log_10_ *P* values in each trait) to identify SNPs forming potentially important clusters even though the individual *P* values might be less significant (Supplementary Table S4 at *JXB* online) as suggested by Verslues *et al.* (2014). We further determined LD blocks harbouring significant SNPs (i.e. –log_10_ *P*>4.0) as regions containing putative candidate genes, which led to a total of 195 genes (Supplementary Table S5 at *JXB* online). For several traits, –log_10_ *P* values and QQ plots suggested relatively weak genetic association (Supplementary Figs S2–S8 at *JXB* online). In the following, we focused on LBS, relative DW, and relative SPW, for which MLM analysis yielded more robust genetic associations taking into account heritability (Supplementary Table S6 at *JXB* online), –log_10_ *P* values, and the QQ plots, where the deviation from the expected values occurred only in the most significant *P* value range.

### LBS

**Fig. 2.**
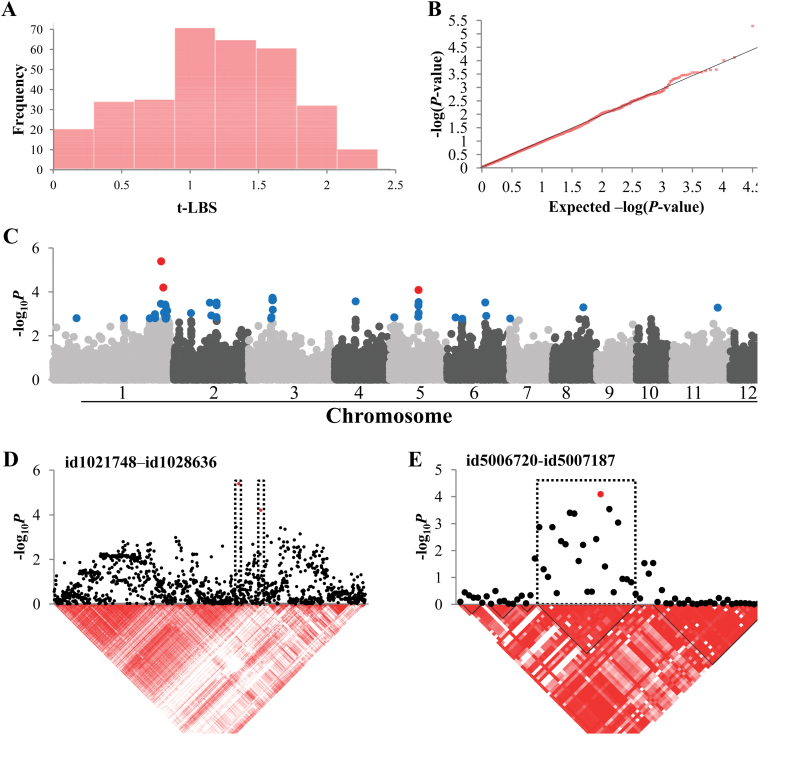
Association mapping result for t-LBS. (A) Frequency distribution of observed t-LBS. (B) QQ plot of expected and observed *P* values. (C) Manhattan plots from association mapping using the MLM. The top 50 SNPs are shown in blue and the SNPs exceeding the significance threshold of *P*<0.0001 are shown in red. (D) The peak region on chromosome 1. (E) The peak region on chromosome 5. In (D) and (E), pair-wise LD between SNP markers is indicated as *D’* values: dark red indicates a value of 1 and white indicates 0. The dotted squares in (D) and (E) denote the LD blocks that contain significant SNPs. (This figure is available in colour at *JXB* online.)

The square-root transformed LBS (t-LBS) ranged from 0.0 to 2.4 (Fig. 2A). The corresponding QQ plot indicated deviation from the expected *P* values only in the most significantly associated markers, thus limiting the possibility of declaring false positives (Fig. 2B). Two distinct peaks were observed on the Manhattan plot when setting the threshold of –log_10_ *P*>4.0 (Fig. 2C). Determining LD blocks on the chromosomal regions where significant SNPs were observed narrowed down the region in which to look for the candidate genes (Fig. 2D, E). On chromosome 1, only one gene was found in the LD blocks. Although not located in the LD blocks, many β-1,3-glucanase genes were identified in the neighbouring region (12 homologues in the surrounding 285kb). One significant SNP marker was located between two β-1,3-glucanase genes: LOC_Os01g71670 (188bp distance) and LOC_Os01g71680 (1.8kb distance). On chromosome 5, the peak was quite sharp, and the flanking LD blocks showed very low –log_10_ *P* values (Fig. 2E). This region contained candidate genes encoding an *EREBP* and a *RING*, which were further characterized by sequencing of contrasting haplotypes, as detailed below. Several other potentially interesting genes were found by analysing LD blocks containing the top 50 SNPs, although most of them did not exceed the threshold of –log_10_ *P*>4.0. One locus on chromosome 2 (associated with id2009675, –log_10_ *P*=3.52) contained a chitinase gene (LOC_Os02g39330) along with four other genes, and a locus on chromosome 3 (associated with 8 SNPs, maximum –log_10_ *P*=3.74) contained a mitogen-activated protein kinase gene (LOC_Os03g17700).

### Relative DW

**Fig. 3.**
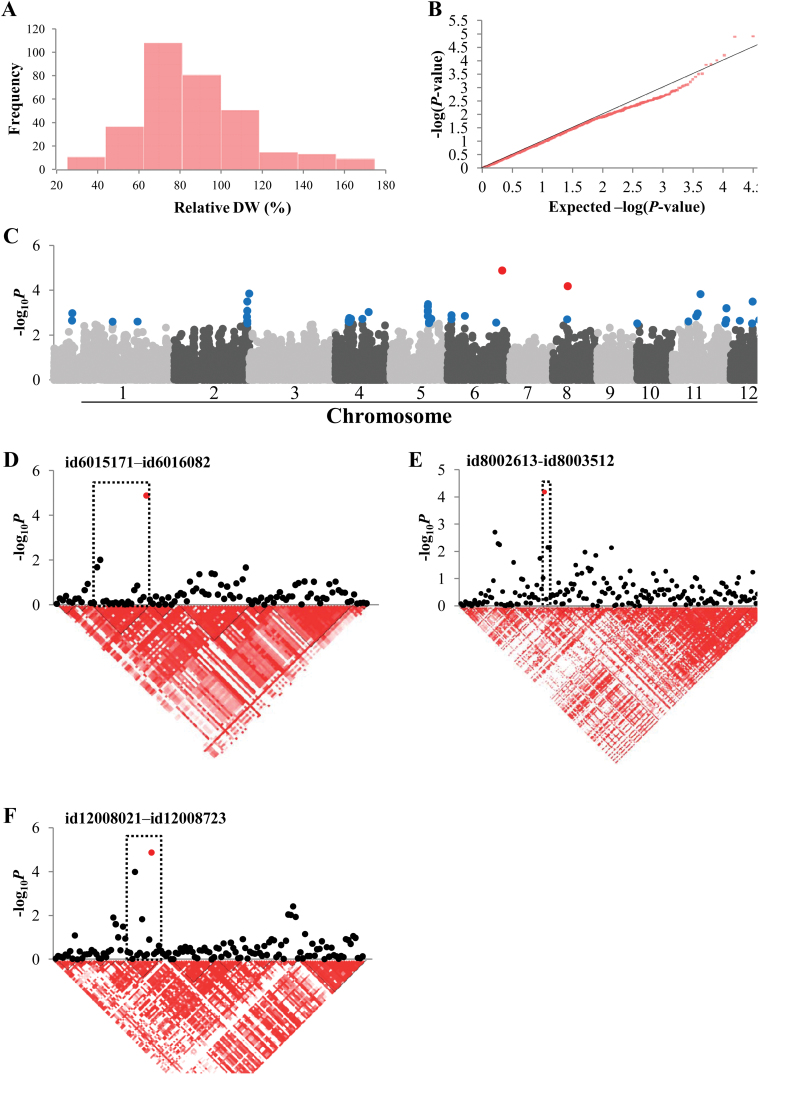
Association mapping result for relative DW. (A) Frequency distribution of observed relative DW. (B) QQ plot of expected and observed *P* values. (C) Manhattan plots from association mapping using the MLM. The top 50 SNPs are shown in blue and the SNPs exceeding the significance threshold of *P*<0.0001 are shown in red. (D) The peak region on chromosome 6. (E) The peak region on chromosome 8. (F) The peak region on chromosome 12. In (D)–(F), pair-wise LD between SNP markers is indicated as *D’* values: dark red indicates a value of 1 and white indicates 0. The dotted squares in (D)–(F) denote the LD blocks that contain significant SNPs. (This figure is available in colour at *JXB* online.)

Relative DW showed a normal distribution (Fig. 3A) and the QQ plot showed deviation from the linear line in the most significant *P* value range (Fig. 3B). Three significant SNPs were located on chromosomes 6, 8, and 12 (Fig. 3C). However, we were unable to identify interesting genes among the ones located within these LD blocks (Figs 3D, E and Supplementary Table S5). We also searched for genes from the LD blocks determined from the top 50 SNPs. A small but sharp peak was observed on chromosome 2. The corresponding LD block (associated with id2016513, –log_10_ *P*=3.85) contained genes such as a putative hexose transporter (LOC_Os02g58530) and a putative sucrose synthase (LOC_Os02g58480), which could be related to carbon metabolism and translocation. Another LD block on chromosome 5 (associated with four SNPs, maximum –log_10_ *P*=2.59) contained several sugar transporters (LOC_Os05g36414, LOC_Os05g36440, LOC_Os05g36450, and LOC_Os05g36700).

### Relative SPW

**Fig. 4.**
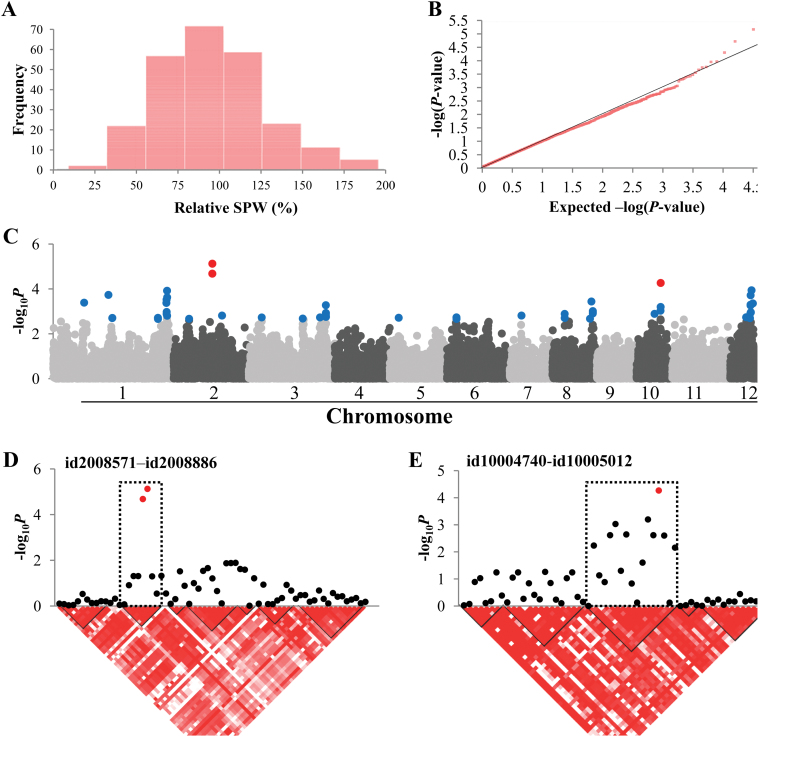
Association mapping results for relative SPW. (A) Frequency distribution of observed relative SPW. (B) QQ plot of expected and observed *P* values. (C) Manhattan plot from association mapping using the MLM. The top 50 SNPs are shown in blue and the SNPs exceeding the significance threshold of *P*<0.0001 are shown in red. (D) The peak region on chromosome 2. (E) The peak region on chromosome 10. In (D) and (E), pair-wise LD between SNP markers is indicated as *D’* values: dark red indicates a value of 1 and white indicates 0. The dotted squares in (D) and (E) denote the linkage blocks that contain significant SNPs. (This figure is available in colour at *JXB* online.)

Relative SPW was distributed approximately normally (Fig. 4A). The –log_10_ *P* values showed deviation from the expected values only in the most significantly associated markers (Fig. 4B), suggesting reliable performance of the MLM for this trait. By applying the threshold of –log_10_ *P*>4.0, we identified three significant SNPs on chromosomes 2 and 10 (Fig. 4C). On both chromosomes, the LD blocks consisted of a relatively small number of SNPs (Figs. 4D, E), and a total of 38 genes were located in the LD blocks containing these three SNPs (Supplementary Table S5). The LD block on chromosome 10 contained 20 genes (excluding retrotransposons and a non-expressed gene), among which six genes had leucine-rich repeat regions including five with a GO annotation of ‘signal transduction’, which could be involved in ozone sensing and triggering downstream reactions.

### Co-localization of candidate loci

We analysed whether some loci were common to more than one trait. By curating the top 50 SNPs from all traits, we found that a total of 28 SNPs were shared in multiple traits (Supplementary Table S4), which suggests pleiotropy of the SNPs or the existence of a closely linked gene (Zhao *et al.* 2011). One of the SNPs (id1027640) affected four traits (relative plant height, relative tiller number, relative TPW, and relative SPW), although no convincing candidate gene was found within the LD block. Some SNPs affected multiple categories of traits, such as leaf cell death and biochemical components (id1026656 and id5000980), leaf cell death and growth parameter (id1027571), and growth parameter and biochemical components (id12005469). These traits sharing common candidate loci were not necessarily correlated with each other (Table 2).

### Candidate gene identification and sequence analysis

**Fig. 5.**
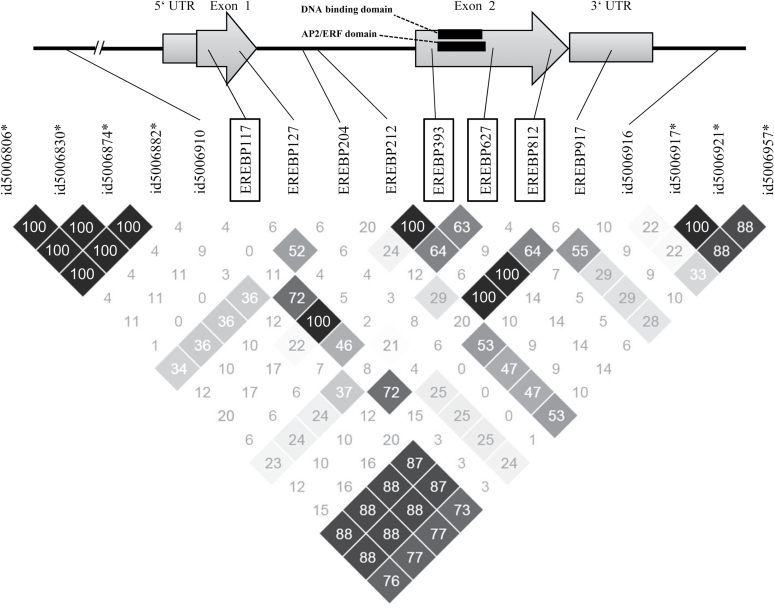
Sequence variation of *EREBP* (LOC_Os05g29810). The polymorphic sites of *EREBP* in 34 accessions are shown together with two adjacent SNP markers and those among the top 50 SNP markers for LBS, which are located within the LD block (shown with asterisks). In the observed polymorphic sites, the number after EREBP indicates the position from the transcription initiation site. The sites within black frames (EREBP117, EREBP393, EREBP627, and EREBP812) cause amino acid substitution/insertion. The matrix shows the *r* ^*2*^ values of each pair-wise comparison of markers and polymorphic sites. Higher values are shown in a darker colour. The number in the square shows the 100-fold value of *r* ^*2*^, which ranged from 0 to 100. The sequence aa 53–111 is a DNA-binding domain, and aa 53–116 is an AP2/ERF domain.

To test the plausibility of candidate genes, we determined sequence variations in contrasting haplotypes, which could be related to functional alterations. We chose the aforementioned locus on chromosome 5, which was identified for LBS, as a target region for the sequencing for the following reasons: (i) leaf cell death is one of the most conspicuous symptoms induced by ozone and is therefore of high importance and physiological interest; (ii) it gave a significant –log_10_ *P* value in the peak region; (iii) LBS had a higher genetic heritability (*h* ^2^_t-LBS_=0.33) than the average heritability from all traits (*h* ^2^_all_=0.27), showing that a larger portion of phenotypic variance for LBS is ascribed to genetic variance (Supplementary Table S6); (iv) the LD block contained a relatively small number of candidate genes; and (v) evidence from other studies strongly supports the role of candidate genes under ozone stress (discussed later). Among the 41 genes contained in this locus (Supplementary Table S5), we selected genes that had informative annotation. Thus, 17 retro- or transposon genes and a further two non-expressed genes were eliminated (Supplementary Table S5). From the remaining 22 genes, we chose genes that were involved in either cell-death pathways or were related to ethylene, which plays a crucial role in inducing cell death (Kangasjärvi *et al.*, 2005), for further characterization by sequencing: an *EREBP* (ethylene-responsive element binding protein, LOC_Os05g29810) and a *RING* (‘really interesting new gene’, LOC_Os05g29710). The EREBP was a transcription factor that contains an ethylene-responsive binding domain. Its closest *Arabidopsis* homologue (At1g53910) is known to regulate oxygen sensing and trigger downstream response (Licausi *et al.*, 2011). The RING protein contained a transmembrane domain and a RING motif. The closest *Arabidopsis* homologue (At5g10380) is related to the induction of pathogen resistance and cell death (Lin *et al.*, 2008). First, we chose contrasting haplotypes for markers surrounding the genes and sequenced the genes. Genome sequences of additional accessions from a public rice genome database were also included in the analysis (Supplementary Fig. S9 at *JXB* online). Analysing these 34 accessions revealed eight nucleotide polymorphisms in the *EREBP* gene (Fig. 5). We assessed the *r* ^*2*^ values between the observed polymorphic sites and those SNPs in the LD block, which were in the top 50 SNPs for LBS. Here, we used *r* ^*2*^ value rather than *D’* value for the assessment of association, since our objective was to get insight into the functional relationship rather than determining LD blocks. The highest *r* ^*2*^ value was observed at a polymorphic site in the intron (position 204 and id5006957, *r* ^*2*^=0.53, *P*<0.0001 by a two-tailed Fisher’s exact test). The polymorphic sites causing amino acid substitutions generally had low *r* ^*2*^ values (average *r* ^*2*^=0.17, highest *r* ^*2*^=0.37, *P*=0.0017). In other words, the observed amino acid substitutions were not closely associated with the significant SNPs detected through GWAS.

**Fig. 6.**
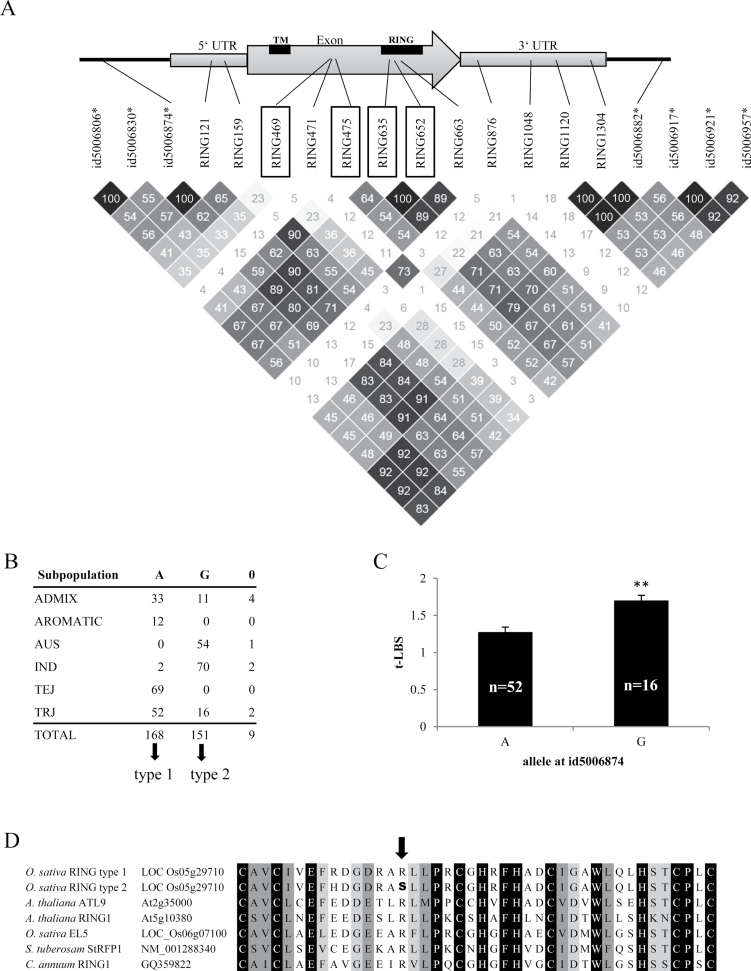
Sequence analysis of *RING* (LOC_Os05g29710) as a candidate gene underlying the peak for t-LBS on chromosome 5. (A) The polymorphic sites of the *RING* gene. The genomic sequences of the *RING* gene from 50 accessions are shown together with two adjacent SNP markers and those among the top 50 SNP markers for LBS, which were located within the LD block (shown with asterisks). In the observed polymorphic sites, the number after RING shows the position from the transcription initiation site. The sites with black frames (RING469, RING475, RING635, and RING652) cause amino acid substitution/insertion. The matrix shows the *r* ^*2*^ values of each pair-wise comparison of markers and polymorphic sites. Higher values are shown in a darker colour. The number in the square shows the 100-fold value of *r* ^*2*^, which ranged from 0 to 100. The sequence aa 29–50 is a transmembrane domain (TM), and aa 133–175 is a zinc-finger motif (RING). (B) Allele frequency of each subpopulation at id5006874. ‘0’ stands for missing data in the original genotyping. Accessions containing A at this position are termed ‘type 1’ and those containing G are termed ‘type 2’. (C) t-LBS in the tropical *japonica* subpopulation. The asterisks indicate that the allele G at id5006874 is associated with a significantly higher t-LBS by Student’s *t*-test (*P*<0.01). (D) Amino acid sequence comparison of the 42 aa RING motif between the rice RING protein and other previously reported cell death or defence reaction-related proteins: *Arabidopsis* ATL9 (Berrocal-Lobo *et al.*, 2010), *Arabidopsis* RING1 (Lin *et al.*, 2008), rice EL5 (Takai *et al.*, 2001), potato StRFP1 (Ni *et al.*, 2010), and pepper RING1 (Lee *et al.*, 2011). Completely conserved amino acids are shown against a black background. Strong groups (as defined by a Pam250 score of >0.5) are shown against a dark grey background and weak groups (as defined by a Pam250 sore of ≤0.5) are shown against a light grey background (Gonnet *et al.*, 1992). An amino acid substitution between type 1 and type 2 (R→S) is shown in bold in the arrowed position.

The *RING* was located near a SNP marker with a high –log_10_ *P* value (id5006874, –log_10_ *P*=3.40) (Fig. 6A). The allele A at the position id5006874 occurred mainly in *aromatic* and temperate *japonica*, and the allele G occurred mainly in *aus* and *indica* subpopulations, while tropical *japonica* subpopulation contained both alleles at a relatively high ratio (Fig. 6B). When conducting association mapping separately for each subpopulation, the same peak at this locus occurred only in the tropical *japonica* subpopulation (Supplementary Fig. S10 at *JXB* online). Therefore, we chose two to five lines from each subpopulation, plus randomly selected additional lines from the tropical *japonica* subpopulation carrying each allele, and sequenced the genomic region of the *RING* gene. We also added rice genome sequences from a public database into the analysis (Supplementary Fig. S11 at *JXB* online). In a total of 50 accessions, nucleotide sequence variation was observed at 12 positions (Fig. 6A). Four of them caused an amino acid substitution or insertion. We determined the correlation between the observed polymorphisms and those among the 50 SNPs that were located within the LD block. Two of the amino acid substitutions (positions 635 and 652) were highly associated with significant SNPs (average *r* ^*2*^ *=*0.70 and 0.65, highest *r* ^*2*^ *=*0.89 and 0.80 respectively, *P*<0.0001 for both positions). Moreover, these two amino acid substitution sites were located in the RING motif, which is crucial for the activity of this protein (Fig. 6A). We classified the accessions according to the allele at id5006874, which showed the strongest association with the amino acid substitutions in the RING motif (Fig. 6B). Type 1 contained allele A at id5006874 and was highly significantly associated with arginine at aa 141 and 147. In contrast, type 2 had allele G at id5006874 and was associated with histidine at the aa 141 and serine at aa 147 (Supplementary Fig. S11). We compared the t-LBS of genotypes carrying the alleles A and G at this position in the tropical *japonica* subpopulation. The allele G was associated with a higher t-LBS than allele A (*P*<0.01) (Fig. 6C). We then obtained the amino acid sequences of previously characterized RING proteins from other species and compared the RING motif sequence with the RING protein in our study. Comparison of the motif showed that a conserved amino acid arginine was substituted by serine in type 2 (Fig. 6D).

## Discussion

We studied the natural variation of rice in response to ozone and identified candidate loci regulating important phenotypes under ozone stress. To the best of our knowledge, this is the first large-scale tolerance screening and GWAS focusing on ozone stress in any crop species. We adopted a target concentration of 60 ppb for the whole season and obtained an average concentration of 63 ppb during the season. This corresponds to an increase of around 25–75% compared with the current average tropospheric ozone concentration (Ainsworth *et al.*, 2012) and is known to cause rice yield reduction by around 14% (Ainsworth, 2008). Additionally, three episodes of acute ozone stress (150 ppb) were applied, which is frequently observed in the early summer season in Asia (Tang *et al.*, 2012). On average, all the growth parameters and SPAD value decreased under ozone stress, while lignin content increased, which is in accordance with previous studies (Shi *et al.*, 2009; Frei *et al.*, 2011; Ainsworth *et al.*, 2013). A slightly higher yield reduction as assessed by TPW in the current study (Table 1) compared with the previous reports might be ascribed to the three episodes of acute ozone stress applied during the season. The substantial genotypic differences observed in ozone response in all phenotypic traits highlight the rich genetic diversity that can be exploited through GWAS. Thus, our fumigation scheme proved optimal to induce a wide range of phenotypic variation and therefore we concluded that this mapping population and the observed phenotypes constitute a powerful resource for association mapping.

The significant positive correlation between growth parameters and yield components suggests source limitations for the grain yield under ozone stress. In other words, carbon assimilation or sugar loading is limiting grain yield, rather than sink limitations such as storage and phloem unloading. Another factor highly affecting the yield could be flowering time. Although we did not measure the flowering time for the whole population, we found a significant negative correlation between relative TPW and the flowering time (i.e. the number of days from transplanting until flowering) recorded in Arkansas (data adopted from Zhao *et al.*, 2011, where the same population was used) (*r*=–0.14, *P*=0.026, Table 2). This negative correlation could suggest that the longer the plants were exposed to ozone before flowering, the more the grain yield was affected. We also found that five SNPs (id1013335, id1013354, id1013362, id1013402, and id1013422) appeared in the top 50 SNPs for both relative TPW and the flowering time recorded in Arkansas, which further implies an effect of flowering time on the grain yield under ozone stress. However, one should be cautious in applying the flowering time recorded elsewhere to our dataset, since this trait is highly affected by day length and temperature sum.

We applied an MLM incorporating both a kinship and PCA matrix to all the phenotypes to avoid false positives, which are likely to result from naïve GLM (Larsson *et al.*, 2013). The QQ plots for t-LBS, relative DW, and relative SPW (Figs 2B, 3B, and 4B) indicated good applicability of the model for these traits (Zhang *et al.*, 2010). For several traits, the MLM might have been too conservative and rendered false negatives, as QQ plots indicated –log_10_ *P* values even below the expected distribution, although Manhattan plots exhibited clearly defined peaks (Supplementary Figs S2–S8). Employing LD blocks to define the genomic regions in which to search for candidate genes has advantages over the fixed-window approach, in which a certain distance from a significant SNP is considered as the region containing candidate genes (Courtois *et al.*, 2013), in terms of elimination of falsely included or excluded genes (Chen *et al.*, 2012). In our study, the candidate regions ranged from <1kb to >1Mb depending on the chromosomal position. This suggests that the resolution of the association mapping depends highly on the LD of the neighbouring regions of the significant SNPs. Since some of the LD blocks harbouring significant SNPs did not contain any annotated gene, this method might have produced some false negatives, or the identified region contained important DNA-binding or gene regulation sites, in which case the causal gene is not directly detected in the LD block (Sur *et al.*, 2013).

Some of the putative candidate genes were involved in pathogen resistance and response. Ozone stress differs from many other abiotic stresses in the sense that the apoplast is the first cell component encountering oxidative stress. Ozone enters the plants through the stomata and produces reactive oxygen species in the apoplast, which induce responses and downstream signals similar to those observed under pathogen attack (Conklin and Barth, 2004), and ultimately lead to programmed cell death (Kangasjärvi *et al.*, 2005), coinciding with the expression of pathogenesis-related (PR) genes (Rao *et al.*, 2000). Thus, the identification of several candidate genes for LBS, which have been characterized in connection with pathogen resistance, appears highly plausible. For example, β-1,3-glucanase and chitinase belong to the glycosyl hydrolase families 19 and 17 and are classified as PR-2 and PR-3 protein, respectively (Brederode *et al.*, 1991). Both have catalytic activity causing degradation of fungal cell walls (Mauch *et al.*, 1988), thereby enhancing the resistance to pathogens (Zhu *et al.*, 1994). Both genes have been recognized as ozone-inducible genes and are proposed to determine ozone sensitivity (Ernst *et al.*, 1992; Street *et al.*, 2011). Since two of the PR proteins were detected near the significantly associated loci based on leaf cell death, our study supports the concept of similarities in pathogen and ozone response.

Another candidate gene possibly associated with visible leaf symptoms is an *EREBP* gene (LOC_Os05g29810), which was found in the LD block containing the significant SNPs on chromosome 5 (Fig. 2E). A low correlation between amino acid substitution/insertion and the significant SNPs (Fig. 5) suggests that the polymorphisms in this gene were not directly associated with the significant GWAS signal. Also, since polymorphisms were not in the functional domain of EREBP, its effect on visible leaf symptoms, if any, would presumably be through post-transcriptional modification or expression level rather than functional alteration of the protein. An even more plausible candidate identified in the peak on chromosome 5 was a *RING* gene (LOC_Os05g29710), encoding an E3 ubiquitin ligase, and is classified as C_3_-H_2_-C_3_ RING protein due to the amino acid sequence of the zinc ion-binding site. This RING protein is part of a large protein family that is emerging as an important factor during pathogen infection and induction of defence reactions (Trujillo and Shirasu, 2010). Amino acid sequence comparison with the homologues showed that one of the amino acid substitutions in the type 2 (which is associated with allele G at position id5006917) is located in the conserved region of the RING motif (Fig. 6D). In many previous studies, amino acid changes leading to functional variance or phenotypic difference occurred in conserved protein motifs (Yamanouchi *et al.*, 2002; Kobayashi *et al.*, 2008). In the case of the RING protein, we assume that this conserved region is crucial for the activity or determines the selectivity of the substrate protein.

In summary, our study demonstrated substantial genotypic variation in ozone tolerance in rice, which provides a rich basis for adaptive breeding. GWAS identified convincing candidate loci based on significant peaks, heritability, LD analysis, and candidate gene identification for LBS. We found a number of novel polymorphisms in an *EREBP* and a *RING* gene, which could be candidate genes controlling visible leaf damage due to their locations in an identified LD block and their annotated functions. These indirect lines of evidence warrant further investigation of these genes and their involvement in ozone tolerance using reverse genetic approaches.

## Supplementary data

Supplementary data are available at *JXB* online.

Supplementary Fig. S1. Subpopulation comparison of all phenotypes.

Supplementary Figs S2–8. Distribution and association mapping results for all phenotypic traits.

Supplementary Fig. S9. Sequence variation of the *EREBP* gene.

Supplementary Fig. S10. Association mapping for square-root transformed leaf bronzing score (t-LBS) in each subpopulation.

Supplementary Fig. S11. Sequence variation of the *RING* gene.

Supplementary Table S1. List of all the phenotypic values and standard deviations.

Supplementary Table S2. Correlation coefficient and *P* value of pair-wise comparison of each phenotype in each subpopulation.

Supplementary Table S3. List of significant SNPs identified through association mapping.

Supplementary Table S4. List of the top 50 SNPs from each phenotype.

Supplementary Table S5. List of genes located within the identified candidate loci for all phenotypes.

Supplementary Table S6. Heritability of each trait.

Supplementary Data

## Abbreviations:

GWAS: genome-wide association study
LBS: leaf bronzing score
LD: linkage disequilibrium
MLM: mixed linear model
QTL: quantitative trait loci
SNP: single-nucleotide polymorphism
SPW: single panicle weight
TKW: thousand-kernel weight
TPW: total panicle weight.
