# Supplementary material for: Genetic dissection of ozone tolerance in rice (Oryza sativa L.) by a genome-wide association study
Source: J Exp Bot. 2014 Nov 4;66(1):293–306. doi: 10.1093/jxb/eru419 (PMC4265164; doi:10.1093/jxb/eru419)
Supplement: Supplementary Data [file supp_66_1_293__index.html]

Genetic dissection of ozone tolerance in rice (Oryza sativa L.) by a genome-wide association study — Genetic dissection of ozone tolerance in rice (Oryza sativa L.) by a genome-wide association study — Supplementary Data 

# Genetic dissection of ozone tolerance in rice (*Oryza sativa* L.) by a genome-wide association study

## Supplementary Data

Data files

**Files in this Data Supplement:**

- Supplementary Data - Supplementary Data
- Supplementary Data - Supplementary Data
