# Supplementary material for: Genetic dissection of ozone tolerance in rice (Oryza sativa L.) by a genome-wide association study
Source: J Exp Bot. 2014 Nov 4;66(1):293–306. doi: 10.1093/jxb/eru419 (PMC4265164; doi:10.1093/jxb/eru419)
Supplement: Supplementary Data [file supp_eru419_jexbot129585_file001.pdf]

Supplementary Figures S1-S11

Genetic dissection of ozone tolerance in rice (*Oryza sativa* L.) by genome wide association study

Yoshiaki Ueda, Felix Frimpong, Yitao Qi, Elsa Matthus, Linbo Wu, Stefanie Höller, Thorsten Kraska, Michael Frei

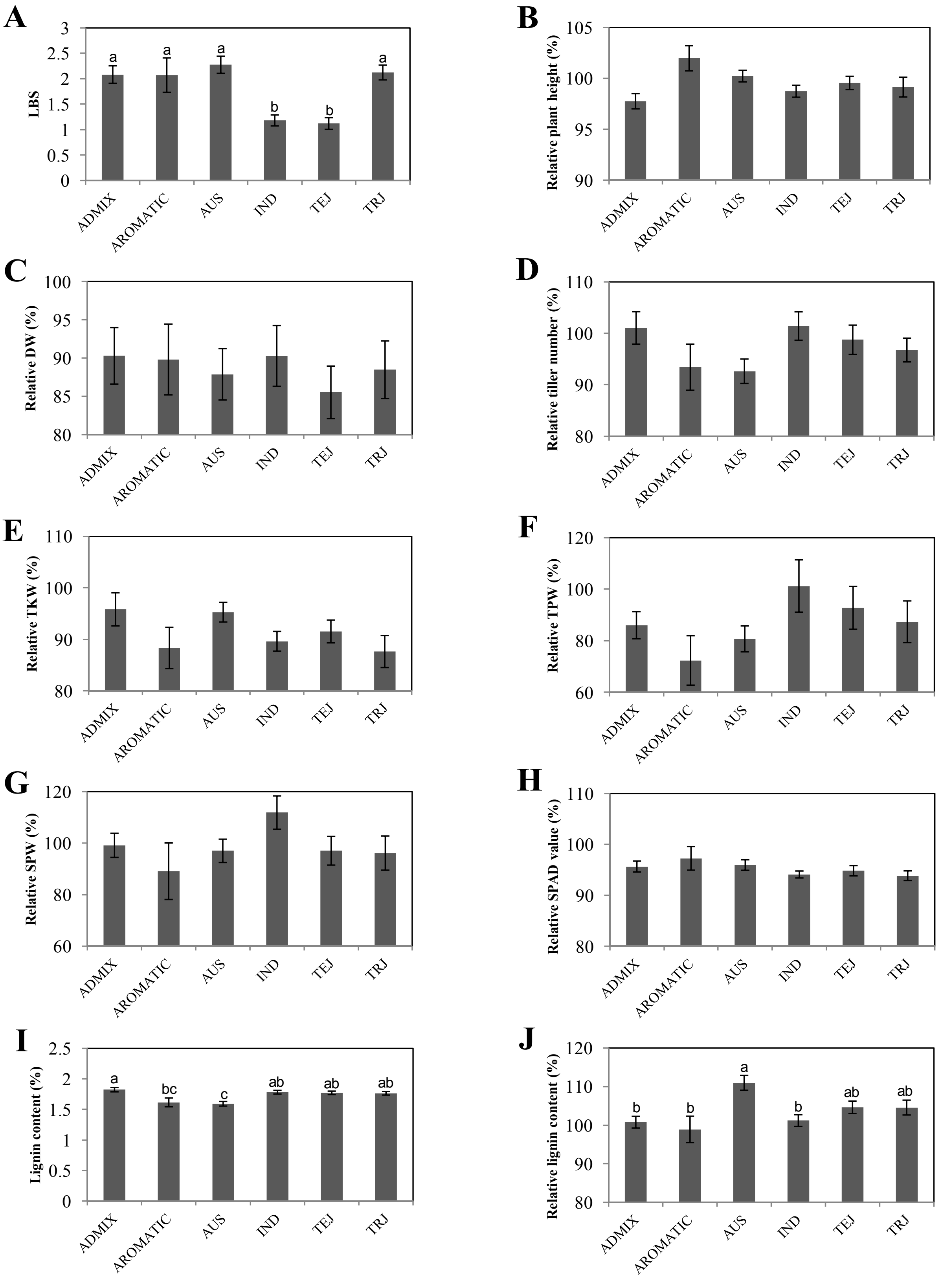

Supplementary Figure S1 Subpopulation comparison of all phenotypes. The whole population was classified into five subpopulations or admixed group as proposed by Zhao *et al.* (2010). Mean values and standard errors are shown. The subpopulations were composed as follows: ADMIX (admixed group, n = 48), AROMATIC (n = 12), AUS (n = 55), IND (*indica*, n = 74), TEJ (temperate *japonica*, n = 69), TRJ (tropical *japonica*, n = 70). In E-G, 63 lines with thousand kernel weight (TKW) less than 10 g (10 ADMIX, 4 AROMATIC, 3 AUS, 26 IND, 4 TEJ and 16 TRJ) were eliminated to evaluate only matured grains. In F and G, lines with high grain shattering (1 ADMIX, 6 AUS and 1 IND) were further eliminated. For

A, I and J, letters above the bars indicate significant differences at $P < 0.05$. All other parameters showed no significant differences. (A) leaf bronzing score (LBS), (B) relative plant height, (C) relative dry weight (DW), (D) relative tiller number, (E) relative TKW, (F) relative total panicle weight (TPW), (G) relative single panicle weight (SPW), (I) relative SPAD value, (H) constitutive lignin content, (J) relative lignin content.

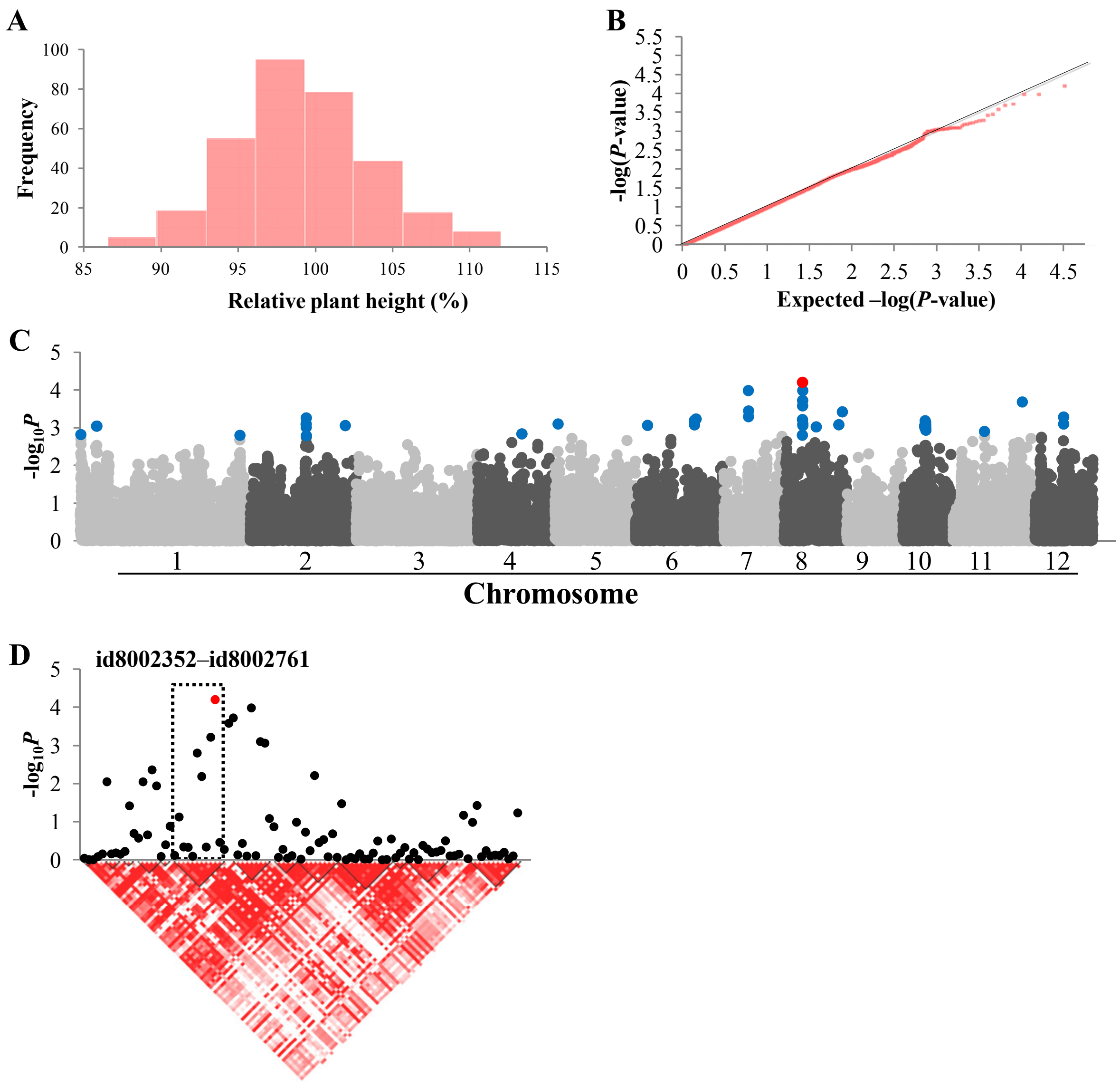

Supplementary Figure S2 Association mapping result for relative plant height. (A) Frequency distribution of observed relative plant height. (B) QQ-plot of expected and observed *P*-values. (C) Manhattan plots from association mapping using Mixed Linear Model (MLM). The top 50 SNPs are shown in blue color and the SNP exceeding the significance threshold of $P < 0.0001$ is shown in red color. (D) The peak region on chromosome 8. In D, pair-wise linkage disequilibrium between SNP markers is indicated as *D'* values: dark red indicates a value of 1 and white indicates 0. The dotted square on D denotes the linkage disequilibrium block which contains the significant SNP.

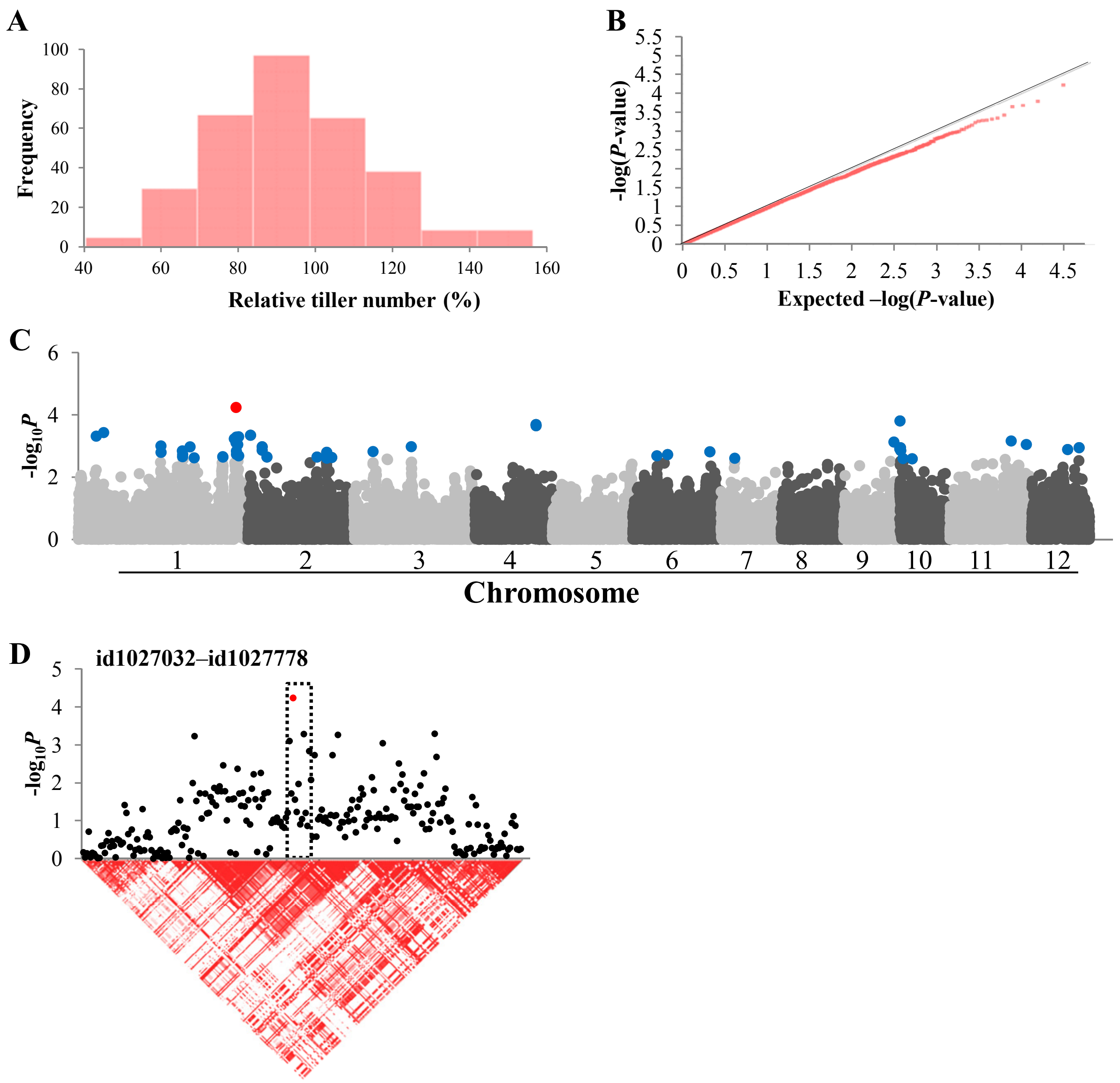

Supplementary Figure S3 Association mapping result for relative tiller number. (A) Frequency distribution of observed relative tiller number. (B) QQ-plot of expected and observed *P*-values. (C) Manhattan plots from association mapping using Mixed Linear Model (MLM). The top 50 SNPs are shown in blue color and the SNP exceeding the significance threshold of $P < 0.0001$ is shown in red color. (D) The peak region on chromosome 1. In D, pair-wise linkage disequilibrium between SNP markers is indicated as *D'* values: dark red indicates a value of 1 and white indicates 0. The dotted square on D denotes the linkage disequilibrium block which contains the significant SNP.

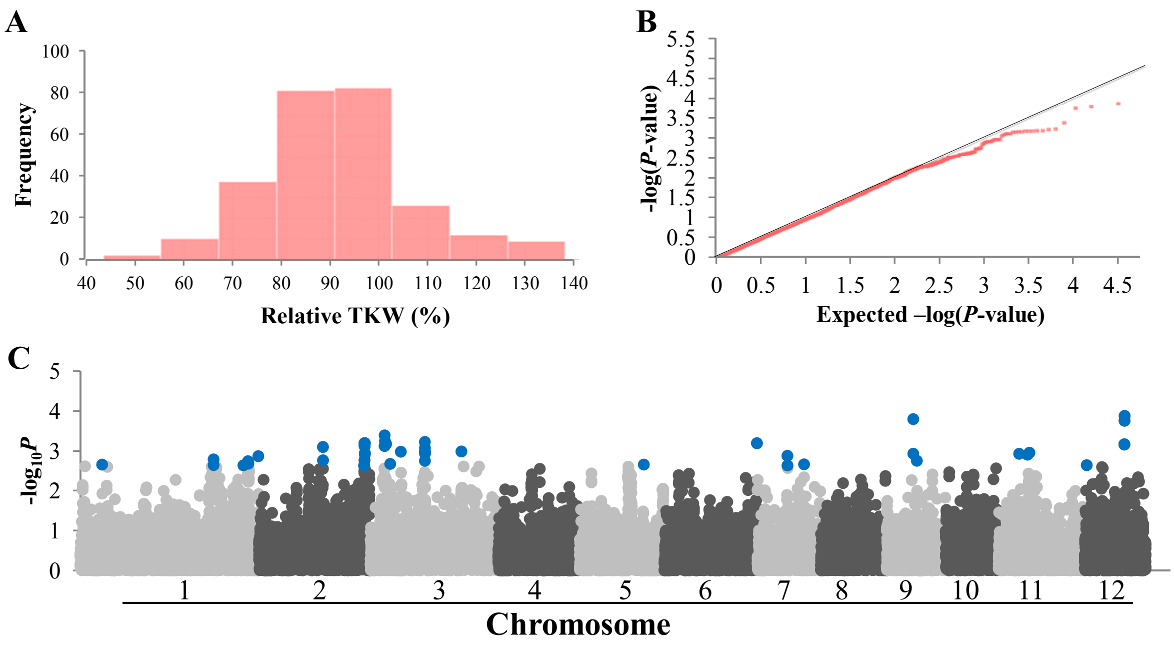

Supplementary Figure S4 Association mapping result for relative thousand kernel weight (TKW). (A) Frequency distribution of observed relative TKW. (B) QQ-plot of expected and observed *P*-values. (C) Manhattan plots from association mapping using Mixed Linear Model (MLM). The top 50 SNPs are shown in blue color.

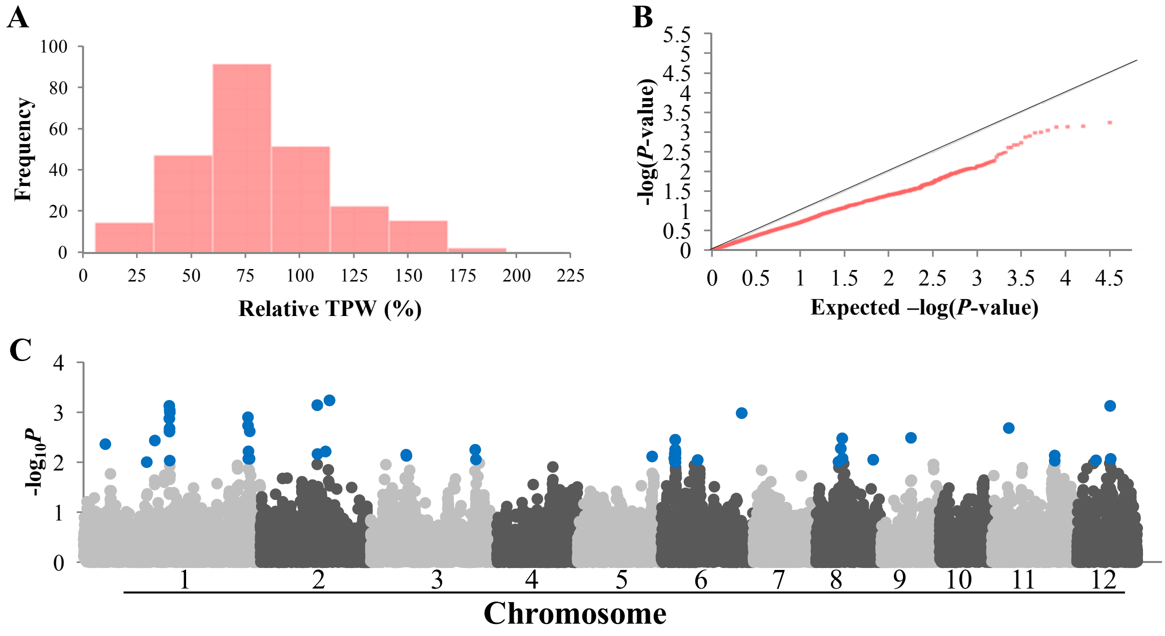

Supplementary Figure S5 Association mapping result for relative total panicle weight (TPW). (A) Frequency distribution of observed relative TPW. (B) QQ-plot of expected and observed *P*-values. (C) Manhattan plots from association mapping using Mixed Linear Model (MLM). The top 50 SNPs are shown in blue color.

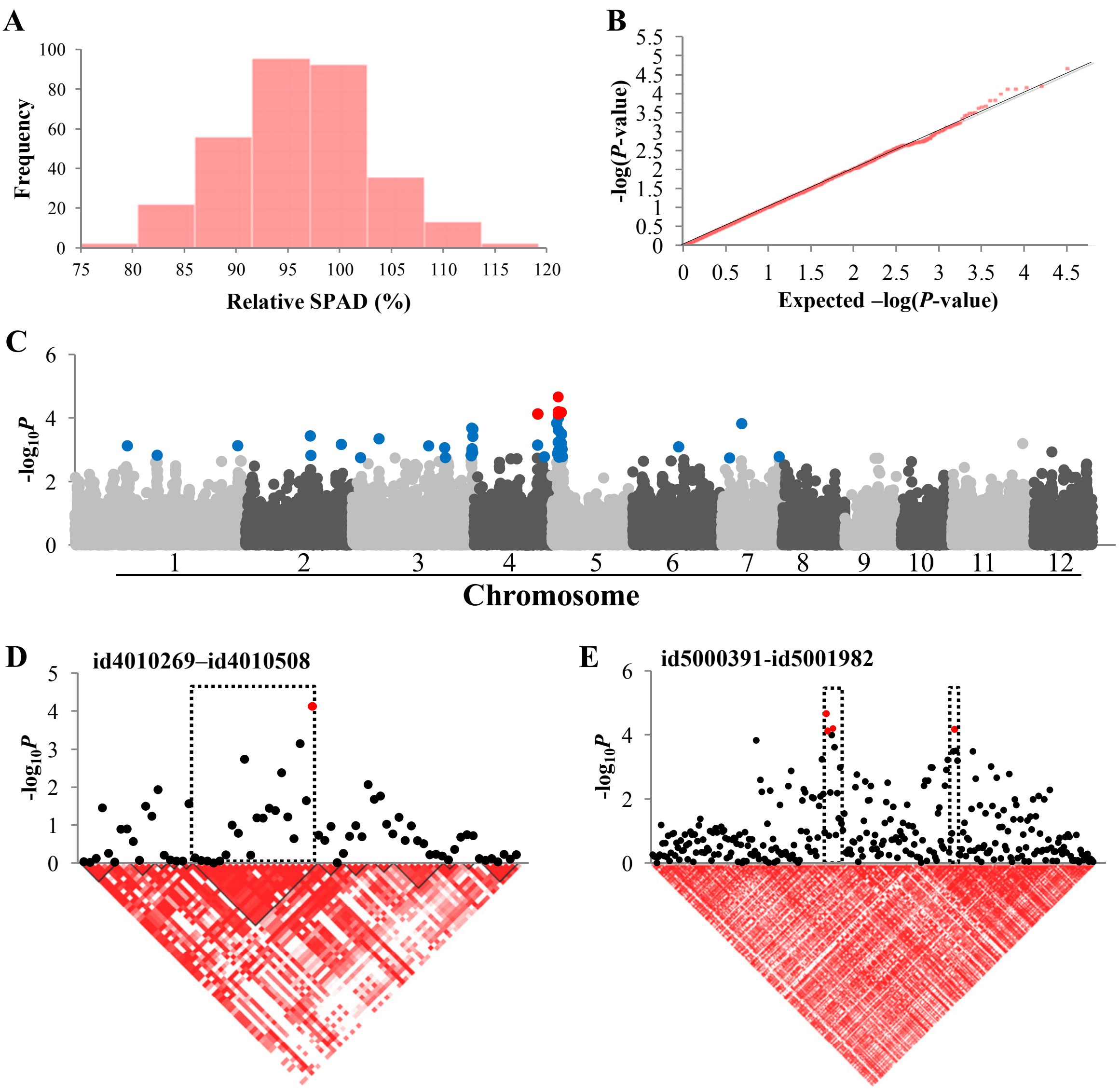

Supplementary Figure S6 Association mapping result for relative SPAD value. (A) Frequency distribution of observed relative SPAD value. (B) QQ-plot of expected and observed *P*-values. (C) Manhattan plots from association mapping using Mixed Linear Model (MLM). The top 50 SNPs are shown in blue color and the SNPs exceeding the significance threshold of $P < 0.0001$ are shown in red color. (D) The peak region on chromosome 4. (E) The peak region on chromosome 5. In D and E, pair-wise linkage disequilibrium between SNP markers is indicated as *D'* values: dark red indicates a value of 1 and white indicates 0. The dotted squares on D and E denote the linkage disequilibrium blocks which contain significant SNPs.

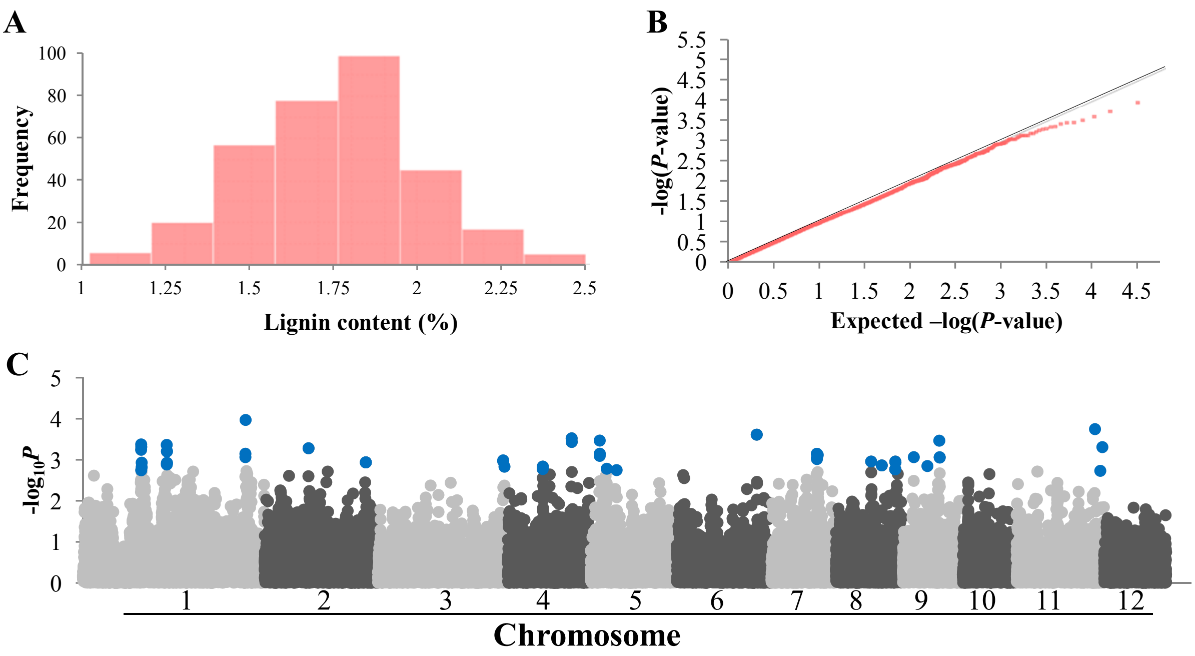

Supplementary Figure S7 Association mapping result for constitutive lignin content. (A) Frequency distribution of observed constitutive lignin content. (B) QQ-plot of expected and observed *P*-values. (C) Manhattan plots from association mapping using Mixed Linear Model (MLM). The top 50 SNPs are shown in blue color.

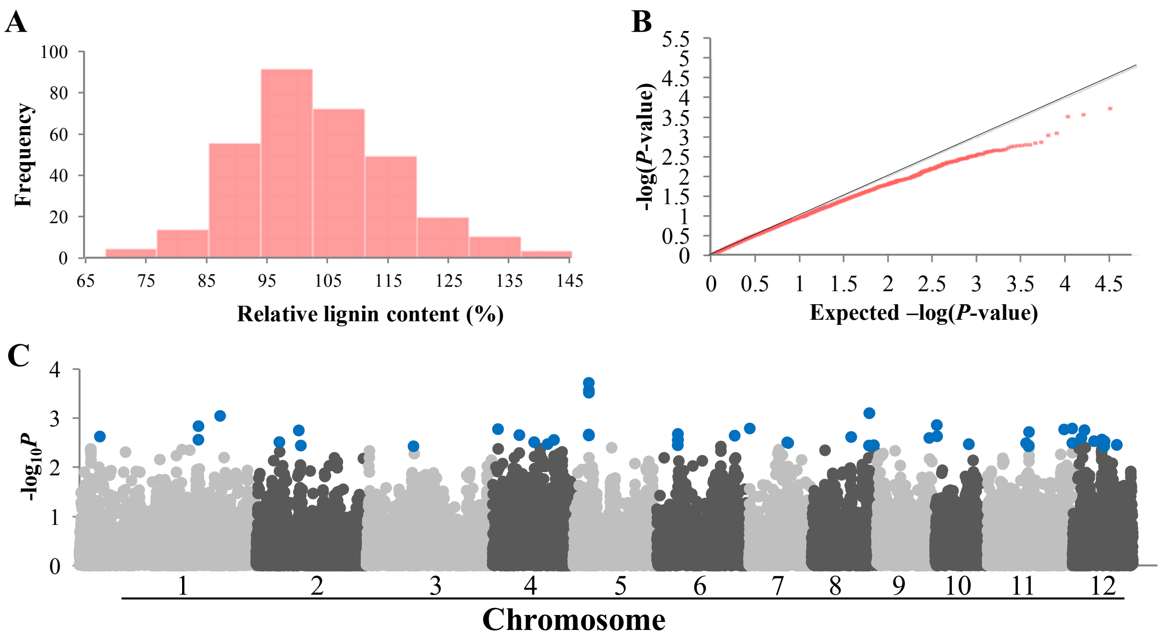

Supplementary Figure S8 Association mapping result for relative lignin content. (A) Frequency distribution of observed relative lignin content. (B) QQ-plot of expected and observed *P*-values. (C) Manhattan plots from association mapping using Mixed Linear Model (MLM). Top 50 SNPs are shown in blue color.

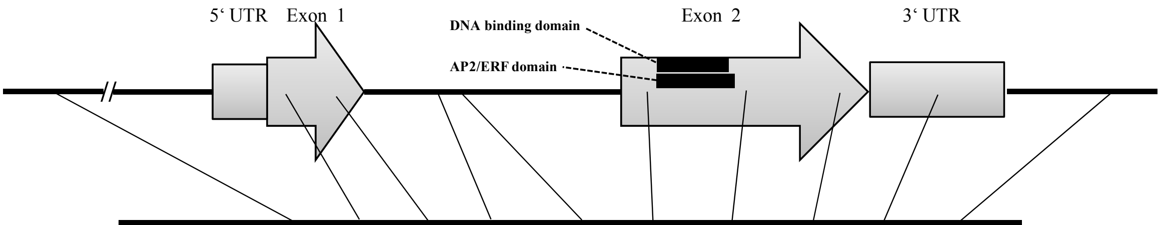

Plant ID	Subpop	id5006910	117	127	204	212	393	627	812	917	id5006916
**639**	**TEJ**	**C**	**G**	**T**	**T**	**T**	**G**	**C**	**- - -**	**A**	**T**
45	ARO	C	G	C	T	T	G	C	GGA	A	C
112	ARO	C	G	C	T	T	G	C	GGA	A	C
318	AUS	T	G	C	C	A	T	T	- - -	C	T
353	AUS	T	G	C	C	A	T	T	- - -	C	T
171	IND	C	A	C	C	T	G	C	- - -	A	T
184	TEJ	C	G	C	T	T	G	C	- - -	A	T
218	ADM	C	A	C	C	T	G	C	- - -	A	T
308	TRJ	C	A	C	C	T	G	C	- - -	A	T
154	TEJ	C	0	T	T	T	0	0	GGA	A	C
41	TEJ	C	G	C	T	T	G	C	GGA	A	C
131	TEJ	C	G	C	T	T	G	C	GGA	A	C
113	TEJ	C	0	0	T	T	0	C	- - -	A	C
32	TEJ	C	G	T	T	T	G	0	- - -	A	T
104	TEJ	C	0	0	T	T	G	C	GGA	A	C
75	TRJ	C	0	0	T	T	0	C	- - -	A	C
17	TRJ	C	G	0	T	T	0	C	GGA	A	C
635	TRJ	C	G	T	T	T	0	C	- - -	A	T
116	TRJ	C	0	T	T	T	G	0	- - -	A	C
165	TRJ	C	0	T	T	T	0	0	GGA	A	C
23	TRJ	C	G	C	T	T	G	C	GGA	A	C
101	TRJ	C	G	C	T	T	G	C	GGA	A	C
93	ARO	C	G	C	T	T	G	C	GGA	A	C
16	ARO	C	G	C	T	T	G	C	GGA	A	C
53	ARO	C	G	C	T	T	G	C	GGA	A	C
107	IND	T	0	T	T	T	0	C	- - -	A	T
102	IND	C	A	C	C	T	G	C	- - -	A	T
71	IND	T	G	C	C	A	T	C	- - -	C	T
3	IND	C	0	T	C	T	0	C	- - -	A	T
61	IND	C	0	0	C	T	0	C	- - -	A	T
105	AUS	C	0	0	T	T	G	C	- - -	A	C
81	AUS	C	A	C	C	T	G	C	- - -	A	T
78	AUS	C	0	T	C	T	G	C	- - -	A	T
50	AUS	C	G	T	T	T	G	C	- - -	A	C
		*	R (17) → H	Syn.	*		A (43) → S	P (121) → S	- (182) → E	*	*

Supplementary Figure S9 Sequence variation of the *EREBP* gene. The genomic sequences of the *EREBP* from a total of 34 lines are shown together with two adjacent SNPs. The amino acid sequence 53-111 is a DNA binding domain, and 53-116 is an AP2/ERF domain. The reference sequence from Nipponbare is shown in bold letters on the top. The possible amino acid substitution in the coding sequence is shown in the bottom row. The amino acid in the reference sequence, its position and the substituted amino acid are shown. “0” stands for missing data. “Syn.” denotes that the SNP causes silent mutation. Asterisks show that the SNP position has been already reported by OryzaSNP project (McNally *et al.*, 2009).

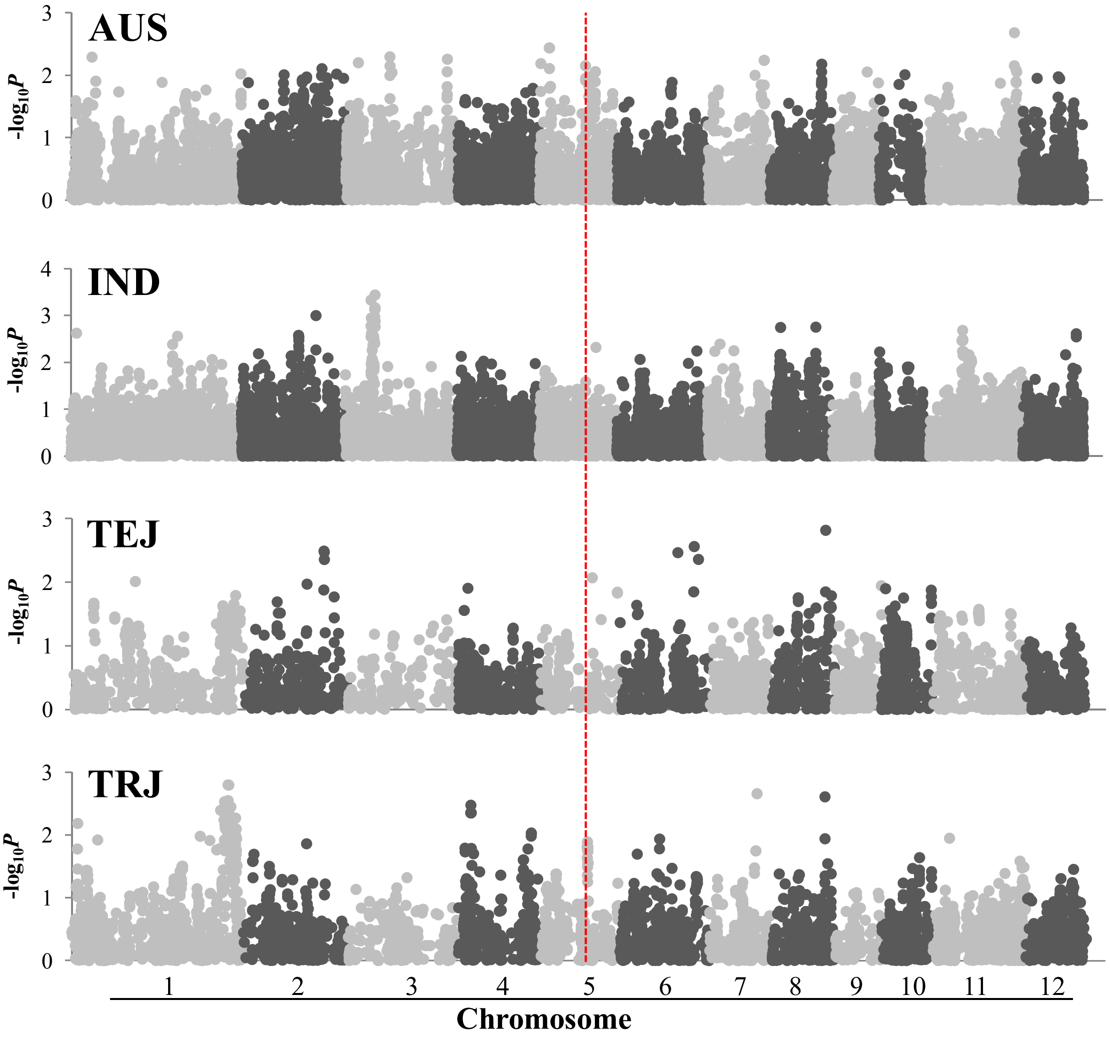

Supplementary Figure S10 Association mapping result in each subpopulation for square-root transformed leaf bronzing score (t-LBS). The subpopulations consisted of AUS (n = 55), IND (*indica*, n = 74), TEJ (temperate *japonica*, n = 69) and TRJ (tropical *japonica*, n = 70). Association mapping was not conducted in ADMIX and AROMATIC subpopulation. The dotted red line on chromosome 5 denotes the position of the *RING* (LOC_Os05g29710).

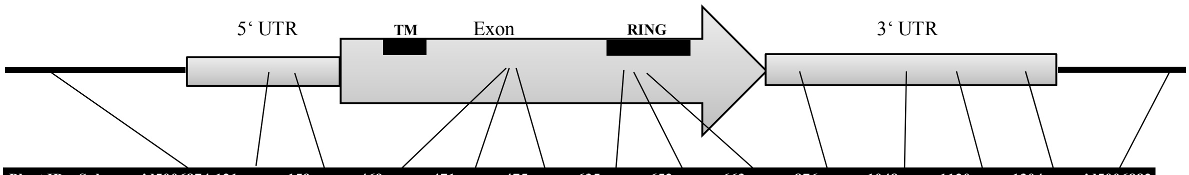

Plant ID	Subpop	id5006874	121	159	469	471	475	635	652	663	876	1048	1120	1304	id5006882
**639**	**TEJ**	**A**	**C**	**G**	**- - - - - -**	**C**	**A**	**G**	**C**	**C**	**C**	**T**	**GT**	**A**	**0**
14	TRJ	A	C	G	- - - - - -	C	A	G	C	C	C	T	GT	A	T
26	TRJ	A	C	G	- - - - - -	C	A	G	C	C	C	T	GT	A	T
45	ARO	A	C	G	- - - - - -	C	A	G	C	C	C	T	GT	A	T
46	TRJ	A	C	G	- - - - - -	C	A	G	C	C	C	T	GT	A	T
112	ARO	A	C	G	- - - - - -	C	A	G	C	C	C	T	GT	A	T
135	TRJ	A	C	G	- - - - - -	C	A	G	C	C	C	T	GT	A	T
184	TEJ	A	C	G	- - - - - -	C	A	G	C	C	C	T	GT	A	T
213	TRJ	A	C	G	- - - - - -	C	A	G	C	C	C	T	GT	A	T
264	ADM	A	C	G	- - - - - -	C	A	G	C	C	C	T	GT	A	T
267	TEJ	A	C	G	- - - - - -	C	A	G	C	C	C	T	GT	A	T
285	TRJ	A	C	G	- - - - - -	C	A	G	C	C	C	T	GT	A	0
390	ADM	A	C	G	- - - - - -	C	A	G	C	C	C	T	GT	A	T
68	ADM	G	G	T	TCGTCG	C	G	A	A	G	C	A	- -	C	C
75	TRJ	G	G	T	TCGTCG	C	G	A	A	G	C	T	- -	C	C
139	TRJ	G	G	T	TCGTCG	C	G	A	A	G	C	A	- -	C	C
166	ADM	0	G	T	TCGTCG	C	G	A	A	G	C	T	- -	C	C
171	IND	G	G	T	TCGTCG	C	G	A	A	G	C	T	GT	A	0
209	IND	0	G	T	TCGTCG	C	G	A	A	G	C	T	- -	C	C
218	ADM	G	G	T	TCGTCG	C	G	A	A	G	C	A	- -	C	C
231	IND	G	G	T	TCGTCG	C	G	A	A	G	C	T	GT	A	T
242	TRJ	G	G	T	TCGTCG	C	G	A	A	G	C	T	- -	C	C
308	TRJ	G	G	T	TCGTCG	C	G	A	A	G	C	A	- -	C	C
318	AUS	G	G	G	TCGTCG	T	A	A	A	G	A	T	- -	C	C
324	AUS	G	G	G	TCGTCG	T	A	A	A	G	A	T	- -	C	C
353	AUS	G	G	G	TCGTCG	T	A	A	A	G	A	T	- -	C	C
635	TRJ	G	G	T	TCGTCG	C	G	A	A	G	C	A	- -	C	C
154	TEJ	A	C	G	0	0	0	G	0	0	C	T	GT	A	T
41	TEJ	A	C	G	0	C	A	G	0	0	C	T	GT	A	T
131	TEJ	A	C	G	0	0	A	G	C	C	C	T	GT	A	T
113	TEJ	A	C	G	0	C	A	0	C	C	C	T	GT	A	T
32	TEJ	A	C	G	TCGTCG	C	A	G	0	0	C	T	GT	A	T
104	TEJ	A	C	G	TCGTCG	C	A	0	0	0	C	T	GT	A	T
17	TRJ	A	C	G	0	0	0	0	0	0	C	T	GT	A	T
116	TRJ	A	C	G	TCGTCG	C	A	G	C	C	C	T	GT	A	T
165	TRJ	A	C	G	0	0	A	0	0	0	C	T	GT	A	T
23	TRJ	A	C	G	TCGTCG	C	A	G	C	C	C	T	GT	A	T
101	TRJ	A	C	G	TCGTCG	C	A	G	C	C	C	T	GT	A	T
16	ARO	A	C	G	TCGTCG	C	A	G	C	C	C	T	GT	A	T
53	ARO	A	C	G	TCGTCG	C	A	G	C	C	C	T	GT	A	T
105	AUS	A	C	G	TCGTCG	C	A	G	C	C	C	T	GT	A	T
50	AUS	A	C	G	TCGTCG	C	A	G	C	C	C	T	GT	A	T
93	ARO	A	C	G	TCGTCG	C	A	G	C	G	C	T	GT	A	T
107	IND	G	G	G	TCGTCG	T	A	0	0	0	C	T	- -	C	C
71	IND	G	G	G	0	0	0	0	0	C	A	T	- -	C	C
61	IND	G	G	T	0	C	A	A	A	0	C	T	- -	C	C
78	AUS	G	G	T	0	C	G	0	0	C	C	T	- -	C	C
81	AUS	G	G	T	TCGTCG	C	G	A	A	G	C	T	- -	C	C
3	IND	G	G	T	0	0	0	G	C	0	C	T	- -	C	C
102	IND	G	G	T	TCGTCG	C	G	0	C	0	C	T	- -	C	C
Amino acid substitution		*	*		- - (86) → SS	*, Syn.	T (88) → A	R (141) → H	R (147) → S	Syn.	*			*	*

Supplementary Figure S11 Sequence variation of the *RING* gene. The genomic sequences of the *RING* from 50 lines are shown together with two adjacent SNPs. The amino acid sequence 29-50 is a transmembrane domain (TM), and 133-175 is a zinc-finger motif (RING). The reference sequence from Nipponbare is shown in bold letters on the top. The possible amino acid substitution in the coding sequence is shown in the bottom row. The amino acid in the reference sequence, its position and the substituted amino acid are shown. “0” stands for missing data. “Syn.” denotes that the SNP causes silent mutation. Asterisks show that the SNP position has been already reported by OryzaSNP project (McNally *et al.*, 2009).
